# The long-term effects of Kerala Diabetes Prevention Program on diabetes incidence and cardiometabolic risk: a study protocol

**DOI:** 10.1186/s12889-023-15392-6

**Published:** 2023-03-22

**Authors:** Tilahun Haregu, T. R. Lekha, Smitha Jasper, Nitin Kapoor, Thirunavukkarasu Sathish, Jeemon Panniyammakal, Robyn Tapp, Kavumpurathu Raman Thankappan, Ajay Mahal, Pilvikki Absetz, Edwin B. Fisher, Brian Oldenburg

**Affiliations:** 1 Baker Heart and Diabetes Institute, Melbourne, Australia; 2 Melbourne School of Population and Global Health, University of Melbourne, Melbourne, Australia; 3 Sree Chitra Tirunal Institute for Medical Sciences & Technology, Trivandrum, India; 4 Christian Medical College, Vellore, India; 5 Department of Family and Preventive Medicine, School of Medicine, Emory University, Atlanta, GA USA; 6 Research Centre for Intelligent Health Care, Coventry University, Coventry, UK; 7 Amrita Institute of Medical Sciences, Kochi, Kerala India; 8 Faculty of Social Sciences, Tampere University, Tampere, Finland; 9 Gillings School of Global Public Health, University of North Carolina, Chapel Hill, USA; 10 School of Psychology and Public Health, La Trobe University, Melbourne, Australia

**Keywords:** Diabetes, India, Kerala, Peer support, Long-term effects, Cardiometabolic, Sustainability

## Abstract

**Introduction:**

India currently has more than 74.2 million people with Type 2 Diabetes Mellitus (T2DM). This is predicted to increase to 124.9 million by 2045. In combination with controlling blood glucose levels among those with T2DM, preventing the onset of diabetes among those at high risk of developing it is essential. Although many diabetes prevention interventions have been implemented in resource-limited settings in recent years, there is limited evidence about their long-term effectiveness, cost-effectiveness, and sustainability. Moreover, evidence on the impact of a diabetes prevention program on cardiovascular risk over time is limited.

**Objectives:**

The overall aim of this study is to evaluate the long-term cardiometabolic effects of the Kerala Diabetes Prevention Program (K-DPP). Specific aims are 1) to measure the long-term effectiveness of K-DPP on diabetes incidence and cardiometabolic risk after nine years from participant recruitment; 2) to assess retinal microvasculature, microalbuminuria, and ECG abnormalities and their association with cardiometabolic risk factors over nine years of the intervention; 3) to evaluate the long-term cost-effectiveness and return on investment of the K-DPP; and 4) to assess the sustainability of community engagement, peer-support, and other related community activities after nine years.

**Methods:**

The nine-year follow-up study aims to reach all 1007 study participants (500 intervention and 507 control) from 60 randomized polling areas recruited to the original trial. Data are being collected in two phases. In phase 1 (Survey), we are admintsering a structured questionnaire, undertake physical measurements, and collect blood and urine samples for biochemical analysis. In phase II, we are inviting participants to undergo retinal imaging, body composition measurements, and ECG. All data collection is being conducted by trained Nurses. The primary outcome is the incidence of T2DM. Secondary outcomes include behavioral, psychosocial, clinical, biochemical, and retinal vasculature measures. Data analysis strategies include a comparison of outcome indicators with baseline, and follow-up measurements conducted at 12 and 24 months. Analysis of the long-term cost-effectiveness of the intervention is planned.

**Discussion:**

Findings from this follow-up study will contribute to improved policy and practice regarding the long-term effects of lifestyle interventions for diabetes prevention in India and other resource-limited settings.

**Trial registration:**

Australia and New Zealand Clinical Trials Registry–(updated from the original trial)ACTRN12611000262909; India: CTRI/2021/10/037191.

## Introduction

Based on recent estimates, there are 537 million people with diabetes globally with 433 million (80.6%) living in low and middle-income countries such as India [1]. The global number of people with diabetes is projected to reach 783 million by 2045. It is estimated that 94% of this increase will occur in low and middle-income countries. Almost 90% of people with undiagnosed diabetes live in low- and middle-income countries [1].

India has the second highest number of people with diabetes (74.2 million), accounting for 1 in 7 of all adults living with diabetes worldwide, and this is expected to increase to 124.9 million by 2045. About 57% of the estimated number of people with diabetes in India are undiagnosed [2]. Partly due to its large population size, India has the second-highest annual number of deaths from diabetes, approximately 0.6 million [1]. In this regard, the prevention and management of diabetes, through community-based approaches need urgent attention [3].

The efficacy, effectiveness, and implementation of lifestyle interventions aimed at preventing the onset of type 2 diabetes have been well-established at least in high-income countries. A review of 38 implementation trials has demonstrated a reduction in type 2 diabetes incidence of between 40–60% [4]. The lifestyle interventions used in these implementation trials have been shown to be more effective when delivered via groups, such as peer support groups. However, most of the trials have been conducted in high-income countries. In addition, there is limited evidence about their effects and sustainability after the active intervention phase.

In this study protocol paper, we briefly discuss the findings of a community-based diabetes prevention program conducted in the Indian state of Kerala, the Kerala Diabetes Prevention Program (K-DPP), from 2013–2016. We also present the rationale to conduct a nine-year follow-up of the original K-DPP participants; the aims, measures to be undertaken, and the analysis plan.

## Summary of Kerala Diabetes Prevention Program (K-DPP) findings at 12 and 24 months

The Kerala Diabetes Prevention Program (K-DPP) is a cluster randomized trial of a group-based and peer-led lifestyle intervention among adults at high-risk for type 2 diabetes in Kerala, India [5]. The intervention was adapted from evidence-based lifestyle interventions implemented in high-income countries, namely, Finland, the United States, and Australia [6]. A total of 60 polling areas (1007 participants) were randomized to the intervention arm (500 participants) or control arm (507 participants) in rural Kerala, India. The intervention was administered over 12 months period. Data on sociodemographic factors, lifestyle or behavioral factors, psychosocial, anthropometric, and biochemical measures were collected at baseline, 12 months, and 24 months. Among K-DPP participants, diabetes had developed in 17.1% of control participants and 14.9% of intervention participants (RR = 0.88; 95% CI: 0.66 -1.16, *P* = 0.36) at 24 months [7]. There was a significant reduction in diabetes incidence among a sub-group of participants with impaired glucose tolerance (IGT) (RR = 0.61, 95% CI: 0.41–0.92) in keeping with findings from the landmark clinical trials conducted on people with IGT [8–10]. The K-DPP program was also associated with a 32% reduction in 10-year cardiovascular disease (CVD) risk in the intervention group compared to the control group (RR = 0.68; 95% CI: 0.47–0.99; *P* = 0.047) among those aged 40 years or more [11]. Effectiveness evaluation has demonstrated significant improvements in certain key CVD risk factors and the physical functioning score of the health-related Quality of life (HRQoL) scale [7]. Moreover, this community-based peer-support lifestyle intervention was found to be cost-effective in individuals at high risk of developing diabetes in India over a 2-year period [11, 12]. The implementation evaluation has shown that the K-DPP program was feasible and acceptable in changing lifestyle behaviors in high-risk individuals [13]. Measures of program reach, adoption, and implementation were excellent for K-DPP. Twenty-nine of the 30 intervention communities delivered all intervention components. Two-thirds of intervention participants attended more than 50% of all peer-led group sessions and program recognition was high among local leaders and champions. There was also very strong community engagement and program sustainability in intervention communities at 24 months with 29/30 groups having conducted other diabetes prevention and health promotion activities in their communities [13].

## Rationale for long-term follow-up of K-DPP participants

### Diabetes incidence and cardiometablic risk

There is limited long-term data on the effect of lifestyle interventions on diabetes incidence and cardiomentabolic risk, especially in low- and middle-income countries. Extended follow-up studies of previous lifestyle intervention studies, such as the Da Qing Diabetes Prevention Study and the U.S. Diabetes Prevention Program, showed benefits in reducing cardiovascular events after many years of follow-up [14, 15]. However, the current evidence is limited and more follow-up studies, particularly from resource-constrained settings, are needed to address this gap. The additional nine years of follow-up in K-DPP enable an assessment of the long-term benefit of lifestyle interventions in terms of reducing diabetes incidence and other cardiometabolic risk. Current and more detailed analyses, including measures of glycemic levels, serum lipids, heart function (ECG), renal function, and the retinal microvaculature, can supplement previous findings. Given the strong study findings and trends at 24 months, it is now important to undertake longterm follow-up to evaluate the program effectiveness, cost-effectiveness, and sustainability of K-DPP.

### Multimorbidity

Diabetes mellitus is one of the main drivers of multimorbidity as it is associated with a large number of risk factors and complications [16]. Consequently, patients with type 2 diabetes mellitus often live with and develop multiple co-occurring conditions with negative impacts on disease management and quality of life [17]. Given the rich baseline and short-term follow-up data from people at high risk of diabetes at enrolment, K-DPP would enable an analysis of progressive development of various multimorbidities, including heart disease and kidney disease, over time in the study population. It also enables evaluation of the association between multimorbidity and glycemic levels in this population.

### Retinal vasculature

Changes in the retinal microvasculature, such as retinal arteriolar narrowing, venular dilatation, and retinopathy, have been associated with various systemic conditions, including impaired fasting glucose, obesity, blood pressure, incident hypertension, incident diabetes, and cardiovascular disease [18]. Retinal vascular calibre has been shown, in prospective cohorts, to be a predictor of type 2 diabetes and impaired fasting glucose. More recent studies and analyses implicate that a wider retinal venular calibre is a marker of chronic hyperglycemia, prediabetes, and microvascular complications of diabetic retinopathy and nephropathy [19]. Wider retinal venular calibre has also been associated changes in body composition, carotid artery disease, incident coronary heart disease, and incident stroke [20]. As compared to retinal venular changes, retinal arteriolar changes are more predictive of hypertension-related comorbidities [21]. Retinal vascular geometry alterations are a novel marker to predict the progression of retinopathy [22]. A follow-up study of K-DPP enables evaluation of the differences in the retinal vasculature between the intervention group and control group who were at high risk for diabetes at enrolment. It will also enable an analysis of anthropological and metabolic parameters that influence retinal vasculature measurements and their differences between groups.

## Methods

### Study aims

The overall aim of this study is to evaluate the long-term effects of the K-DPP on diabetes incidence and cardiometabolic risk at nine years following participant recruitment.

The specific objectives are:

1. To estimate the effects of K-DPP on diabetes incidence and cardiometabolic risk at nine years
2. To assess retinal microvasculature, microalbuminuria, and electrocardiogram (ECG) abnormalities and their association with incident diabetes and cardiometabolic risk factors
3. To evaluate the cost-effectiveness and return on investment of the K-DPP at nine years
4. To assess the sustainability of community engagement, peer support, and other related community activities after nine years

### Study design and setting/context

The current study is the nineth-year follow-up assessment of the K-DPP. The protocol paper for the original trial has been published elsewhere [5]. K-DPP was a cluster RCT conducted in 60 polling areas (clusters, electoral divisions with geographical boundaries) of *Neyyattinkara* taluk (subdistrict) in Trivandrum district, Kerala state, India. Participants were screened and recruited in 2013 and the 12-month intervention was completed in 2013–2014 [23, 24]. The study area has been affected by floods during monsoon season in more recent years [25]. particularly the study areas of Kattakada and Neyyatinkara. The continuum of care for chronic diseases during this period was affected adversely due to the impact on access to care and increased risk of other medical conditions. The recent COVID-19 pandemic has also severely affected the study area with cases peaking in October 2020, May 2021, and August 2021 [9]. Additionally, travel restrictions during lockdowns during COVID pandemic posed discontinuities in NCD care [26].

### Study population

The study population for K-DPP is adults 30–60 years of age who were at high risk for diabetes based on the Indian Diabetes Risk Score (IDRS ≥ 60) [27] at the time of enrolment. Of the total 1007 participants who were enrolled in K-DPP, 47% were females. Each peer group selected two peer leaders (one male and one female) from among the participants with the assistance of the intervention team. The 60 peer groups each with 10 to 23 participants had an approximately equal number of males and females. The peer leaders were trained by the intervention team consisted of an intervention manager and an intervention assistant to deliver the intervention [7].

### Sample size

#### Primary outcomes

Based on the available data at 12 and 24 months, we anticipate more than 90% of K-DPP participants at baseline can provide complete and valid data for all key measures underlying diabetes incidence and 10-year CVD risk predicted using the Framingham Risk Score [28] at nine years. The proportion of participants (≥ 40 years of age at baseline) at high-rosk of CVD (with a predicted 10-year CVD risk greater than 20% [29]) is expected to be 34.5% in the control arm (*n* = 348) versus 17.9% in the K-DPP arm (*n* = 337) after nine years, assuming a linear increasing trend overtime based on our data so far. The power to detect this difference is more than 97% when accounting for an intra-cluster correlation of 0.02 at a two-sided 5% level of significance.

#### Secondary outcomes

Loss to follow-up has been very low so far, i.e., < 5% at 24 months and a 7-year cohort study conducted in the same region had a follow-up rate of 92% [30]. Assuming missing data (i.e., no lost outcome data due to reasons such as loss-to-follow-up or no blood sample) reaches 18% in the control arm and 22% in the intervention arm, 415 and 390 participants in the control and intervention arm, respectively are predicted to provide data at 9-years of follow-up. With the sample sizes of 415 and 390 participants in the control and intervention arms respectively, we shall be able to detect an effect size (i.e., the absolute difference in means divided by standard deviation) in other continuous outcomes (e.g., anthropometric, BP, microvascular and cholesterol measurements) of 0.23 with 80% power at the two-sided 5% significance level, when accounting for an intra-cluster correlation of 0.02. Power was derived using power *twoprop* and *clustersampsi* in Stata software (version 17 StataCorp LP, College Station, TX, USA).

### Intervention

Detailed description of the cultural adaptation and development of the K-DPP intervention has been published previously [6, 31]. Briefly, K-DPP involved a 12-month peer-support program that consisted of 15 group sessions: an introductory session delivered by the K-DPP team; two education sessions conducted by local experts; and 12 sessions delivered by trained lay peer leaders. Group sessions were held in the local community in a convenient neighbourhood facility and at convenient time during weekends. The peer group sessions aimed to increase physical activity, promote healthy eating habits, maintain appropriate body weight by balancing calorie intake and physical activity, tobacco cessation, reduce alcohol consumption, and ensure adequate sleep. In the control communities, an educational booklet concerning information about diabetes and its risk factors, as well as standard advice about lifestyle change were provided. After the implementation of interventions, a 12-month and 24-month follow-up assessment was conducted [5, 6].

### Data collection instruments and measurements

Most of the measurements undertaken in previous rounds of assessment are also undertaken in this follow-up assessment (Table 1). A detailed description of these measurements has been published [5]. Newly added measurements include an assessment of the retinal microvasculature, ECG, multimorbidity questionnaire, urine albumin, serum creatinine, sedentary behavior, and community engagement/sustainability questions. The measurements used in all rounds of assessment are summarized in Table 1 below.

**Table 1:** Measurements included in the four time points of assessment

Variable	Component	Measurement tools/questions	Baseline	12 months	24 months	9-Years
Socio-demography	Age, sex, marital status, education, religion, occupation, household size, and monthly household expenditure		✓	✓	✓	✓
Behavioral measures	Tobacco use	WHO STEPS questionnaire [32]	✓	✓	✓	✓
	Alcohol use	WHO STEPS questionnaire [32]	✓	✓	✓	✓
	Physical activity	Global Physical Activity Questionnaire [33]	✓	✓	✓	✓
	Sedentary behavior	Sitting and screen time	X	X	X	✓
	Sedentary behavior	Time spent in front of a screen [34]	✓	✓	✓	✓
	Diet	Food Frequency Questionnaire [35]	✓	✓	✓	X
Diabetes knowledge	Barriers to healthy eating	Scale designed for trial	✓	✓	✓	✓
	Barriers to PA	Adapted from Booth et al. [36]	✓	✓	✓	✓
	Self-efficacy	Adapted Schwarzer and Renner [37]	✓	✓	✓	✓
Psychosocial measures	Depression	Patient Health Questionnaire-9 [38]	✓	✓	✓	✓
	Stress	Chronic stress scale used in MESA study [39]	✓	✓	✓	✓
	Anxiety	General anxiety disorder scale [40]	✓	✓	✓	✓
	HRQoL	Short Form-36 [41]	✓	✓	✓	✓
	Social support	ENRICHD social support scale [42]	✓	✓	✓	✓
	Life satisfaction	Single question: Satisfaction with life	✓	✓	✓	✓
Medical history	Medical history	CVD and diabetes-related medications	✓	✓	✓	✓
	Family history	Updated family history of disease	✓	✓	✓	✓
	Multimorbidity	Adapted from the WHO SAGE questionnaire [43]	X	X	X	✓
Clinical measures	Anthropometry	Waist circumference (Seca measuring tape)	✓	✓	✓	✓
		Height (Seca stadiometer)	✓	✓	✓	X
		Weight and body composition (TANITA)	✓	✓	✓	✓
		Bioimpedance (TANITA)	✓	✓	✓	✓
	Blood pressure	Omron automatic blood pressure monitor	✓	✓	✓	✓
Biochemical measures	Pathology	Glycaemic control (fasting plasma glucose)	✓	✓	✓	✓
		Glycaemic control -OGTT	✓	✓	✓	X
		Glycaemic control (HbA1c)	✓	✓	✓	✓
		Lipid profile (TBC, HDL, LDL, triglycerides)	✓	✓	✓	✓
		Serum creatinine	X	X	X	✓
		Urine albumin	X	X	X	✓
Assessment of heart conditions		ECG	X	X	X	✓
Retinal microvasculature		Retinal imaging measures(Retinal architecture, arteriolar and venular diameters, and tortuosity) [44]	X	X	X	✓
Mortality		Status and self-reported causes of death	X	X	X	✓
Economic Evaluation	Healthcare utilization	Direct and indirect costs associated with outpatient and inpatient healthcare services, sources of financing, and time away from work due to ill health + SF-36	✓	✓	✓	✓
Community engagement	Sustainability	Sustainability – continued engagement in peer groups/activities; contacts with peer leaders; intention to continue engagement	X	X	X	✓

*WHO* World Health Organization, *SF-36*  Short Form 36, *MESA* Multi-Ethnic Study of Atherosclerosis, *OGTT*  Oral Glucose Tolerance Test, *TBC*  Total Blood Cholesterol, *HDL* High Density Lipoprotein, *LDL* Low Density Lipopotein

### Data collection procedures

#### The research team

The research team consist of three research nurses, a research associate, and a project manager. The team attended a five-day training on the study objectives, data collection tools, and data collection procedures. They have also receive additional training on data entry using RedCap, Retinal Imaging, ECG, and TANITA measurements. Each of these training has practical sessions.

*Phase I (Survey) data collection*: In this phase, the research team collects Questionnaire-based data including weight and BP measurements and biochemical (blood and urine) samples. Members of the research team visit the houses of participants to collect data and samples. The samples are be transferred to A laboratory accredited by the National Accreditation Board for Laboratories(NABL) for testing.

*Phase II (community-based clinics) data collection*: In this phase, we identify fixed facilities in which all the equipment can be placed and organize community-based clinics at these community facilities on specific dates and invite participants to have their measurements (height, body composition analyzer (TANITA), refractometry, retinal measurements, and ECG)undertaken.

### Strategy to reach out to participants after nine years

During the preparation phase of the K-DPP follow-up study, participants were contacted via phone to check their availability and willingness to participate in the follow-up study. Unique K-DPP identification numbers and contact details of the study participants and local resource persons (LRP) from the previous assessments were used to reach to participants. For the survey data collection, the study team visited the homes of participants. Repeat visits are being organized if participants are not available on the first visit. The participants who denied consent are not part of the current evaluation. Concerted efforts to reach out to the missing participants (The participants who are not available at the time of data collection and whose contact details could not be traced) are being made. For community-based clinics, participants are invited to undertake the measurements.

### Long-term effectiveness outcomes

The key study outcomes are the incidence of diabetes and CVD risk at nine years after the intervention. Other primary outcomes include Blood pressure, weight, HbA1c, triglycerides, SF-36, and tobacco. Additionally, body mass index, fat percent, muscle mass, waist circumference, diastolic blood pressure, Fasting Plasma Glucose, lipid profile (total cholesterol, LDL cholesterol, and HDL cholesterol), current alcohol use, diet, and physical activity will be assessed. New measures of pre-clinical retinal microvasculature, heart function and renal disease are also being assessed. We are also considering multimorbidity as a secondary outcome in this follow-up study.

#### Anthropometry and behavioral measures

Anthropometry (weight, total body fat percent, muscle mass, waist circumference) and Blood Pressure are measured using standard protocols. Self-reported physical activity and sedentary behavior are estimated using the Global physical activity questionnaire (GPAQ). Fruit and vegetable consumption, alcohol use, and tobacco use are assessed using the WHO STEPs Questionnaire.

Two TANITA machines (model SC330) used in the previous rounds of assessment were sent to the designated national TANITA service center for recalibration. Weight and body composition parameters will be measured using these TANITA body composition analyzers while the participant is standing still without footwear, with one foot on each side of the scale, facing forward, and arms at their side.

#### Retinal vasculature measurement

A CANON CR-2/Plus/AF RX non-mydriatic retinal camera is being used to obtain retinal images. Two images of each eye are being taken (macular centred and disc centred), following a standard protocol. Image quality will be continuously monitored throughout the study. Images will be graded using fully automated software providing measures of arteriolar and venular diameter and tortuosity. The refractive status of the eyes are being assessed using aCanon RK-F2 Autorefractometer.

#### ECG

We are using ECG equipment (KardioScreen-1612) to acquire the report that is being asssed to determine whether the ECG waves and intervals are normal or pathological. A standard operating procedure adapted from the American College of Cardiology guidelines [45]. KardioScreen records 12-lead ECG and works along with an application on Android Tablet/Mobile. ECGs are being recorded in a standardized way with the patient in the supine position, and leads will be placed. The patient’s clinical history is recorded along with the ECG. The application records ECG readings and stores them on the Cloud. A real-time automatic AI-based software (iMedrix) is fed by the recorded ECGs, and data classification and analytical monitoring of heart health parameters is being done. This includes heart rate [14], PR, QT/QTc, QRS duration, and the specific ECG patterns that occur based on electrophysiological changes in a diseased heart.

#### Biochemistry

All biochemical measurements are following standard procedures. Samples are being centrifuged within 30 min of collection, and transported with dry ice to a nationally accredited laboratory. Plasma glucose are being measured with the Hexokinase method on a COBAS 6000 analyzer, with kits supplied by Roche Diagnostics. The quality of plasma glucose measurements from the proposed laboratory is good for the K-DPP trial; the intra-class correlation coefficient approached 1.0. HbA1c is being measured by High-Performance Liquid Chromatography on a D-10 BIORAD analyzer and lipids by enzymatic methods on COBAS 6000, kits supplied by Roche.

#### Microalbuminuria

The random urine samples are being processed using Roche/Hitachi Cobas 501 analyzer system by Immunoturbidimetric assay with Roche reagent cassettes. Before running the samples, quality control procedures are being followed using Roche precinorm PUC and Precipath PUC. Besides, a monthly Biorad EQAS urine chemistry programme including microalbumin and creatinine is also being followed. Cobas 501 system automatically calculates microalbumin 24-h, random and microalbumin and creatinine ratio.

### Economic evaluation

The economic evaluation involves cost-effectiveness analysis of the intervention from both the health system and societal perspectives. The key outcome measure will be Quality Adjusted Life Years (QALY), which will be estimated using the utility values (SF-6D) derived from the SF-36 survey form. Costs being assessed include both direct medical and non-medical costs and indirect (lost days of productivity) costs associated with the intervention. These are be based on information on staff inputs by duration and type and resource use collected during the study. Any additional “knock-on” effect on hospital admissions or outpatient service use is being quantified and costed for each study participant. We are calculating direct costs to the health service and patients from health facility records, project financial accounts, and participant responses to a survey including questions on health service use and spending. These analyses will be based on updated estimates for health facility costs and personal costs Personal costs to individuals will be collected as part of the questionnaire administered to study participants at nine years. These include transportation costs and out-of-pocket spending for health services use, especially for medications and will capture how the individual financed these costs (e.g., borrowing, asset sales, dis-savings). Any indirect costs to patients arising from being absent from work due to the intervention or illness are also being estimated. Savings from lowered health service use associated with the intervention are subtracted from intervention costs to derive incremental costs at the 9-year mark.

### Program sustainability

We are assessing three key features of K-DPP program sustainability at nine years from intervention using a structured interview with K-DPP participants. This assessment focuses on the past 12 months preceding the assessment. The five main areas of assessment for program sustainability are:

1. Participation in group/educational sessions and/or peer group activities
2. Contact with peer leaders and/or group members
3. Current and future interest to participate in group sessions/activities
4. Recent efforts made in relation to improving lifestyle factors
5. Factors that would encourage or hinder participation in peer group activities

### Data management plan

#### The survey, biomedical and clinical data

Data are being entered by the research team into the RedCap database at SCTIMST servers. The database has validation checks for the values. Skip patterns are included using branching logic functions. Each team member double-checks the data s/he entered before synchronization. The project manager checks the entered data on weekly basis. Any data entry issues are rectified against the questionnaire. At 25%, 50%, 75%, and 100% of the data entry, the project manager re-checks 10% of the data for quality. After data cleaning is complete, a master copy of the dataset will be created and backed up. All data transformations (derivation of new variables) will be documented in the data dictionary. All hardcopy and electronic data will be properly secured at SCTIMST offices and servers. Data sharing will be based on the guidelines stipulated by the Data Management and Publications committee of the project.

#### Retinal data

Image quality is being continuously monitored throughout the study. Initially, the day’s images were reviewed on site and feedback was provided to the photographer(s) immediately and appropriate measures have been taken to ensure that the images are captured accurately. During subsequent camps / data acquisition during the study, a few images per day are being sent for evaluation and any concerns while taking the photograph will be sent along with the images and a feedback is sent with comments and suggestions within 48 h. In case of any emergency, the contact persons can be contacted for problem resolutions.

Images are being stored on the laptop computer and backed up at the end of each day onto the external hard drive and institutional repository. Each back-up is being labelled by the date of back-up. Apart from quality control images, image transfer is be performed at the end of the study by physical transfer of an external USB hard drive. The best image for each eye will be uploaded into the software for gradeability for each view and then all measurement indices will be obtained.

The data from grading of retinal images will be entered into the RedCap database of the K-DPP Follow-up study. The Participant ID will be used to link the retinal imaging data and the rest of the data from the K-DPP Follow-up study. All data cleaning will be conducted in the same database.

### Statistical analysis

The two co-primary outcomes are diabetes incidence and CVD risk. The analyses will observe the intention-to-treat principle. A generalized estimating equation [46] model with appropriate link function and robust standard errors to account for clustering at polling areas will be fitted to estimate the relative risk (and 95% CI) for the co-primary outcomes at nine years. The sensitivity of the results to assumptions on the missing outcome data underlying this model will be examined using the method of multiple imputations to handle missing data and a pattern-mixture model.

To evaluate the treatment effect on continuous outcomes, a repeated measures mixed-effects linear regression model, adjusting for the baseline measures, will be fitted. Any skewed continuous outcome variable may be transformed before fitting this model. The model will specify treatment, time-point, and treatment by time-point interaction as fixed effects. Random effects will be specified for polling areas, to account for the clustered study design, and for participants, to account for correlation between the repeated measurements on the same participant. The estimated treatment effect (e.g., the absolute difference in means between the treatment arms) at nine years and two-sided 95% CI will be obtained. Binary other outcomes (e.g., diabetes) will be analyzed using GEE models similar to that of the co-primary outcomes.

The retinal image measurements will be compared between the treatment and control groups on the impact of a lifestyle modification program on pre-clinical changes in the retinal microvasculature and its correlation with cardiometabolic risk factors. Retinal imaging measures will include measures retinal architecture such as arteriolar and venular diameters and tortuosity.

The economic evaluation will consist of a cost-effectiveness analysis that will compare the incremental costs and 9-year effects (QALYs) between the study groups. Generalized linear models (GLM) with gamma family and log link components will be used to estimate the incremental costs and QALYs and the results will be presented as incremental cost-effectiveness ratios (ICERs) [47]. QALY models will be adjusted for the baseline utility values (SF-6D). Uncertainty in the cost and QALY estimates will be estimated by non-parametric bootstrapping method [48] and the results will be graphically presented as cost-effectiveness planes and cost-effectiveness acceptability curves. Several sensitivity analyses will be carried out based on different assumptions about the costs, using different discount rates (3%, 5% and 10% per annum), and multiple imputation to assess the sensitivity of the main results due missing cost and QALY data.

Quantitative descriptive analyses will use key indicators—participation in group activities, maintenance of contact with peer leaders, interest to participate in group activities and recent effors made to improve lifestyle factors—to examine the extent of program sustainability from program participants perspectives. Statistical analyses will characterize the relationships among these indicators and the patterns of maintenance of other outcome measures over time.

### Ethics approvals

For the K-DPP Follow-up study, we obtained Ethics approval from the Institutional Review Board of SCTIMST (ID: SCT/IEC/1349/APRIL-2019) and the Alfred Human Research Ethics Committee (ID: 463/21). Local approvals from HMSC and State Health Departments have been obtained before the start of data collection. A participant information sheet that describes the objectives of the study and the study procedures are being provided to participants along with the informed consent form. Participants provided written informed consent for each phase of the study.

## Discussion

This paper describes the protocol for the nine-year follow-up of a cluster randomized controlled trial of a peer-led lifestyle intervention program – the Kerala Diabetes Prevention Program—to reduce the incidence of type 2 diabetes among individuals at high risk of developing type 2 diabetes at baseline. The study will generate evidence that could establish the long-term effectiveness, cost-effectiveness, and sustainability of lifestyle intervention programs to prevent diabetes in India and other resource-constrained settings. Moreover, evidence on the progressive development of various multimorbidities over time among people with or at high risk of diabetes would inform policies and practice in the prevention and management of diabetes in India and other low-resource settings.

## Trial status

Currently following-up participants.

## Data Availability

Not applicable.
